# Decreased Type I Interferon Production by Plasmacytoid Dendritic Cells Contributes to Severe Dengue

**DOI:** 10.3389/fimmu.2020.605087

**Published:** 2020-12-17

**Authors:** Vinit Upasani, Carolina Scagnolari, Federica Frasca, Nikaïa Smith, Vincent Bondet, Axelle Vanderlinden, Sokchea Lay, Heidi Auerswald, Sothy Heng, Denis Laurent, Sowath Ly, Veasna Duong, Guido Antonelli, Philippe Dussart, Darragh Duffy, Tineke Cantaert

**Affiliations:** ^1^Immunology Unit, Institut Pasteur du Cambodge, Institut Pasteur International Network, Phnom Penh, Cambodia; ^2^Department of Medical Microbiology and Infection Prevention, University of Groningen and University Medical Center Groningen, Groningen, Netherlands; ^3^Laboratory of Virology, Department of Molecular Medicine, Affiliated to Istituto Pasteur Italia—Cenci Bolognetti Foundation, Sapienza University of Rome, Rome, Italy; ^4^Translational Immunology Lab, Institut Pasteur, Paris, France; ^5^Virology Unit, Institut Pasteur du Cambodge, Institut Pasteur International Network, Phnom Penh, Cambodia; ^6^Kantha Bopha Children Hospital, Phnom Penh, Cambodia; ^7^Epidemiology and Public Health Unit, Institut Pasteur du Cambodge, Institut Pasteur International Network, Phnom Penh, Cambodia

**Keywords:** dengue virus, plasmacytoid dendritic cell, myloid dendritic cells, interferon stimulated genes, simoa immunoassay, type I interferon

## Abstract

The clinical presentation of dengue virus (DENV) infection is variable. Severe complications mainly result from exacerbated immune responses. Type I interferons (IFN-I) are important in antiviral responses and form a crucial link between innate and adaptive immunity. Their contribution to host defense during DENV infection remains under-studied, as direct quantification of IFN-I is challenging. We combined ultra-sensitive single-molecule array (Simoa) digital ELISA with IFN-I gene expression to elucidate the role of IFN-I in a well-characterized cohort of hospitalized Cambodian children undergoing acute DENV infection. Higher concentrations of type I IFN proteins were observed in blood of DENV patients, compared to healthy donors, and correlated with viral load. Stratifying patients for disease severity, we found a decreased expression of IFN-I in patients with a more severe clinical outcome, such as dengue hemorrhagic fever (DHF) or dengue shock syndrome (DSS). This was seen in parallel to a correlation between low IFNα protein concentrations and decreased platelet counts. Type I IFNs concentrations were correlated to frequencies of plasmacytoid DCs, not DENV-infected myloid DCs and correlated inversely with neutralizing anti-DENV antibody titers. Hence, type I IFN produced in the acute phase of infection is associated with less severe outcome of dengue disease.

## Introduction

Dengue virus is an arthropod-transmitted viral disease of the genus Flavivirus (family Flaviviridae). The virus is endemic in more than 100 countries, and currently half of the world population is at risk of infection (1). An estimate made in 2010 approximates 390 million dengue infections per year, of which 96 million had clinical manifestations (2). DENV strains are distinguished into four antigenically distinct serotypes, DENV-1 to DENV-4, which co-circulate in the same hyperendemic areas (3). Dengue virus infection can cause a spectrum of clinical presentations ranging from asymptomatic infection to more severe forms of disease such as dengue hemorrhagic fever (DHF) and dengue shock syndrome (DSS). Pathogenesis of severe dengue is complex and probably results from an exacerbated host immune response. Most primary infections are mild and are likely to provide lifelong protection against the infecting serotype. In contrast, heterotypic secondary DENV infection (with a DENV serotype distinct from the primary infecting serotype) is the greatest risk factor for severe disease (4, 5). Manifestations of severe disease coincide with a drop in viral load and an inflammatory cytokine storm of unknown origin (6).

Type I IFNs (IFN-I), mainly represented by IFNα and -β, are cytokines involved in orchestrating innate and adaptive immune responses against viral infections. However, systemic and excessive productions of IFN-I are known to have detrimental effects to the host, as they correlate with inflammation, immunosuppression and homeostatic dysfunction (7). IFN-I responses are mainly triggered through the binding of viral RNA to RIG-I-like receptors (RLRs), RIG-I, and MDA5, along with endosomal Toll-like receptor 3 (TLR3) and TLR7 (8). IFN regulatory factor (IRF) 3 and 7 are primary transcriptional factors downstream of RLRs/TLRs signaling, and promote IFN-I response induction during DENV infection (9, 10), albeit IRF7 plays a more important role than IRF3 in stimulating the early production of IFN-I (11). In order to balance IFN-I defense with inflammatory damage, FOXO3, a member of the forkhead family of transcription factors, has been recently identified as a negative regulator of IRF7 transcription (12).

DENV evasion strategies interfering with IFN-I production have been identified from *in vitro* studies. Indeed, DENV overcomes IFN-I-defense mechanisms in primary human immune cells, which appear to have a key role in modulating pathogenesis (13). However, the *in vivo* direct implications of the IFN-I evasion mechanisms adopted by DENV have, so far, been poorly explored, mainly due to the technical challenges to detect low circulating levels of different IFN-I proteins (14–19). Although almost all nucleated cells can produce IFN-Is in response to viral infection, dendritic cells (DCs), and mainly plasmacytoid DCs (pDC) are a major source of IFN-I (20–22). Myeloid DCs (mDC), on the other hand, have been shown to be the predominant target of DENV infection in the peripheral blood (23–25). DENV infection impairs DC function including IFN-I signalling (26), leading to less efficient priming of DENV-specific adaptive immune responses (24). Frequencies of mDCs and pDCs seem to be reduced in the blood of DENV-infected patients during acute phase of disease, but the correlation with disease severity is less clear (27–29).

Here, we aimed to perform a comprehensive evaluation of type I IFN responses during the earliest phase of disease, within 96h of fever onset in a cohort of Cambodian children. We conducted an integrated analysis of expressed genes related to IFN-I signaling in PBMCs, determination of plasma IFNα/β by ultrasensitive digital ELISA and extensive DC subset phenotyping in patients stratified for infection history and disease severity. We show a robust type I IFN response induced after DENV infection compared to healthy individuals. IFNα and IFNβ protein concentrations correlated to DENV viral load and the presence of circulating pDCs in DENV-infected patients. Increased amount of both IFN-I-related transcripts and increased IFNα/β protein concentrations were detected in patients with mild disease compared to severe dengue patients classified as DHF/DSS. In parallel, we observed a correlation between IFNα protein concentrations and platelet counts, a hallmark of severe infection, indicating that a strong and early type I IFN response is beneficial after DENV infection. Finally, IFN-I responses were inversely correlated to anti-DENV antibody titers.

## Materials and Methods

### Ethics Statement

Ethical approval for the study was obtained from the National Ethics Committee of Health Research of Cambodia. Written informed consent was obtained from all participants or the guardians of participants under 16 years of age prior to inclusion in the study.

### Patient Recruitment

Blood samples were obtained from hospitalized children (≥ 2 years) who presented with dengue-like symptoms at the Kanta Bopha Hospital in Phnom Penh, Cambodia. The time-point for collection of blood samples was within 96 h of fever onset at hospital admittance. Patients were classified according to the WHO 1997 criteria upon hospital discharge (30) as dengue fever (DF) or dengue haemorrhagic fever/dengue shock syndrome (DHF/DSS). In addition, age and sex-matched healthy donors were recruited from a cluster-based investigation in Kampong Cham province. Demographics and laboratory parameters of the patients included for each type of analysis as described below can be found in **Table 1**.

**Table 1:** Demographic data and clinical parameters comparing the studied populations.

	Total	DF	DHF/DSS	Healthy donors
**Number of samples**	115	84	31(22/9)	43
**Age (mean** ± **SD)**	8.4 ± 3.9	8.6 ± 3.9	7.5 ± 4.1	9.4 ± 4.2
**M/F ratio**	0.9	1,00	0.6	1,69
**Weight (mean, kg)**	25.7	25,9	22.1	28.4
**Height (mean, cm)**	120.0	121,5	117.3	124.3
**Temperature (mean, °C)**	37.9	37,9	37.6	N/A
**Hematocrit (%)**	39.4	39.3	39.2	
**Platelets (x 10^9/L)**	103.1	102.3	96.7	
**Day of fever at inclusion (mean, range)**	3.5 (1–4)	3.4 (1–4)	3.5 (2–4)	
**Day of fever at discharge (mean, range)**	6.3 (2–10)	5.2 (2–8)	7.2 (5–10)	
**DENV RT-qPCR +**	107	82	30	
**Viral load (copies/ml) (median, IQR)**	19200 (2450–874750)	31200 (795–981250)	9360 (5850 -160250)	
**DENV-1**	33	37	3	
**DENV-2**	67	44	23	
**DENV-3**	0	0	0	
**DENV-4**	7	8	0	
**NS1+ RDT**	77	61	16	
**DENV IgM MACS ELISA**	48	33	13	
**Secondary infection (%)**	74	72	77	

Patients are characterized according to the WHO 1997 criteria. DENV serotype and viral load were determined by RT-qPCR. Primary or secondary infection was determined based on HIA results on acute and convalescent samples. N/A, not applicable; IQR, interquartile range; DF, dengue fever; DHF, dengue hemorrhagic fever; DSS, dengue shock syndrome; RDT, rapid diagnostic test.

### Laboratory Diagnosis

Plasma specimens obtained from patients were tested for presence of DENV using a nested RT-qPCR at the Institut Pasteur in Cambodia, the reference laboratory for arboviral diseases in Cambodia (31). NS1 and anti-DENV IgM/IgG positivity was determined using rapid diagnostic test (combo test for NS1 and IgM/IgG detection, SD Bioline Dengue Duo kits from Standard Diagnostics – Abbott, Chicago, IL, USA). Anti-DENV IgM was measured with an in-house IgM-capture ELISA (MAC-ELISA), as previously described (32). Samples from patients positive for DENV were further tested with hemagglutination inhibition assay (HIA) (33) at admittance and discharge to determine primary/secondary DENV infection as per WHO criteria (30).

### Foci Reduction Neutralization Assay

Foci reduction neutralization test (FRNT) is used as the gold standard to determine the level of neutralizing antibodies against different viruses. Neutralizing antibody titers against the infecting DENV serotype were determined by FRNT assay using reference DENV reference strains: DENV-1 Hawaii (GenBank: **AF425619**), DENV2 New Guinea C (GenBank: **AF038403**), and DENV-4 H241 (GenBank: **AY947539**). The FRNT was performed on samples obtained at discharge, on average 3 days after hospital admittance in order to reach sufficient high titers for detection (**Table 1**). Briefly, Vero-CCL cells (ATCC CCL-81) cultured in Dulbecco’s modified Eagle medium (DMEM,; Sigma-Aldrich, St. Loius, MO, USA) supplemented with 10% fetal bovine serum (FBS; Gibco, Wattham, MA, USA) and seeded in 96-well plates. Heat-treated plasma samples were serially diluted, mixed with equal volume of DENV and incubated for 1 h at at 37°C. Afterwards, plasma-virus mixtures were transferred onto cells in the respective wells. After 1 h of incubation at 37°C, the mixture was replaced with 1.8% carboxymethyl cellulose (Sigma-Aldrich) and incubated at 37°C at 5% CO_2_. At 2–3 days post infection, cells were fixed and stained as described (34) using DENV serotype specific polyclonal hyperimmune ascites fluids (Institut Pasteur du Cambodge). The titer of neutralizing antibodies was expressed as FRNT90, i.e. the plasma dilution at which a 90% reduction in the number of virus-induced foci is observed, and was calculated by regression analysis (GraphPad Software, Inc., La Jolla, CA, USA).

### Dendritic Cell Subset Phenotyping

PBMCs were isolated from dengue positive patients and age-matched healthy donors using Ficoll-Histopaque (GE Healthcare, Chicago, IL, USA) density gradient centrifugation and stored in liquid nitrogen. The samples were thawed in RPMI supplemented with 10% FBS, washed with sterile PBS and then surface stained using the following antibodies: HLA-DR Alexa Fluor 488 (clone L243), Lineage cocktail APC [clone UCHT1 (CD3), clone HCD14 (CD14), clone HIB19 (CD19), clone 2H7 (CD20), clone HCD56 (CD56)], CD11c PE (clone 3.9), CD123 BV510 (clone 6H6) (all from BioLegend, San Diego, CA, USA). Samples were acquired on BD FACS Canto II (BD Biosciences, Franklin Lakes, NJ, USA) using FACSDiva software (BD Biosciences) and were analyzed by FlowJo v10.0 software (BioLegend, San Diego, CA, USA).

### RNA Extraction, Reverse Transcription and qPCR for Gene Expression Analysis

RNA extraction was done from PBMCs of DENV-positive patients and healthy controls by QIAGEN RNeasy Micro Kit (Qiagen, Hilden, Germany) as per manufacturer’s instructions. cDNA was synthetized from extracted RNA using SuperScript II Reverse Transciptase kit (Invitrogen, Carlsbad, CA, USA). Quantitative real-time PCR for IFN-α2, IFN-β, IFN-I receptor subunits (IFNAR1 and IFNAR2), IRF7, and FOXO3 was carried out with the LightCycler 480 instrument (Roche, Basel, Switzerland). Primers and probes for each gene were added to the Probes Master Mix (Roche, Basel, Switzerland) at 500 and 250 nM, respectively, in a final volume of 20 μl. The housekeeping gene β-glucuronidase was used as an internal control. Gene expression values were calculated by the comparative ΔΔCt method. The primers and probe were assayed on demand and were purchased from Integrated DNA Technologies (Coralville, IA, USA). The list of primers and probes is as follows: IFN-α2 (Hs.PT.58.24294810.g), IFN-β (Hs.PT.58.39481063.g), IFNAR1 (Hs.PT.58.20048943), IFNAR2 (Hs.PT.58.1621113), IRF7 (Hs.PT.58.24613215.G), and Foxo3 (Hs.PT.58.5045552.g).

### Single-Molecule Array Digital ELISA

IFNα and IFNβ protein plasma concentrations were quantified by Simoa digital ELISA developed with Quanterix Homebrew kits (Quanterix, Billerica, MA, USA) as previously described (35). For the IFNα assay, the 8H1 antibody clone was used as a capture antibody after coating on paramagnetic beads (0.3 mg/ml), the 12H5 clone was biotinylated (biotin/antibody ratio = 30/1) and used as the detector, and recombinant IFNα17 (Peprotech, Cranbury, NJ, USA) was used as the standard. For the IFNβ assay, the 710322-9 IgG1, kappa, mouse monoclonal antibody (PBL Assay Science, Piscataway, NJ, USA) was used as a capture antibody after coating paramagnetic beads (0.3 mg/ml), the 710323-9 IgG1, kappa, mouse monoclonal antibody (PBL Assay Science) was biotinylated (biotin/antibody ratio = 40/1) and used as the detector antibody, and recombinant protein (PBL Assay Science) was used to quantify IFNβ concentrations. The limit of detection (LOD) of the IFNα and IFNβ assays were 0.005 fg/ml and 0.05 pg/ml, respectively. Plasma IFNα was below the assay detection limit in one patient with DHF/DSS and was excluded from the analysis.

### Statistical Analyses

Statistical analyses were done using GraphPad Prism 7.00 software (GraphPad). Since the data included in the study did not pass the criteria for normality as determined using D’Agostino-Pearson normality test, non-parametric Mann-Whitney U test was used to compare data between two groups. Values were expressed as median and interquartile range. Correlations between groups which did not pass the criteria for normality as determined using D’Agostino-Pearson normality test were calculated by Spearman analysis. For all analyses, p values less than 0.05 were considered statistically significant.

## Results

### Patient Population

In total, we included 115 dengue-positive paediatric patients admitted at Kantha Bopha Children’s hospital, Phnom Penh, during the acute phase of infection with an onset of symptoms less than 96 h before admission for IFN-I analysis and DC phenotyping (**Supplementary Figure 1A**). These patients were classified for immune history (primary/secondary DENV infection) and severity according to WHO 1997 criteria (30). Of these, 84 were classified as DF, 22 as DHF and 9 as DSS patients (**Table 1**). Of the DF patients, 15.2% were undergoing a primary DENV infection whereas 70.6% went through were undergoing a secondary infection, and immune status could not be determined in 14.2% of the cases based on the obtained HIA data (30). 77% of DHF and DSS patients encountered a secondary infection while infection status could not be determined for 23% of the patients (**Table 1**). Of the 107 patients included with measurable viremia, 31% were infected with DENV-1, 63% with DENV-2, and 7% with DENV-4. No DENV-3 infected individuals were included as DENV-3 was not present in circulation at time of patient recruitment. Viral load as determined by qRT-PCR was not significantly different between DF and DHF/DSS patients (**Table 1**, **Supplementary Figure 1B**). As viremia is dependent on immune status, infecting serotype, day of fever and possibly comorbidities, we observe a wide variability in viral load in children with DF and DHF/DSS (36, 37). As frequencies of immune cells and the immune response can vary from day to day during acute DENV infection (38), it is important to emphasize that not only were all patients recruited within 96 h of fever onset, there was no difference in day of fever at inclusion between DF and DHF/DSS patients (**Table 1**). In addition, 43 age and sex-matched healthy donors were recruited from a cluster-based investigation in Kampong Cham province (**Table 1**).

### Increased IFNα/β Protein Concentrations During Acute Dengue Infection

**Figure 1:**
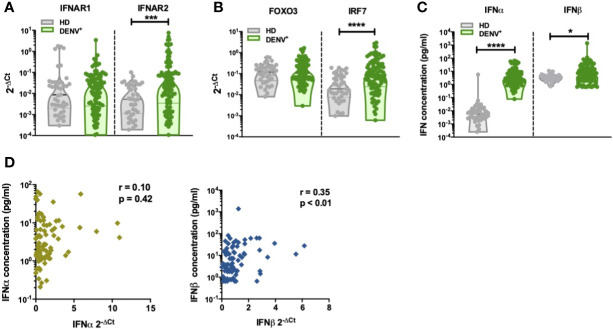
Increased production of IFN-I in DENV patients **(A)**. The expression of IFNα receptor genes IFNAR1 and IFNAR2 was quantified by RT-qPCR and reported as 2^-ΔCt^ values in PBMCs isolated from healthy donors (n=41) and hospitalized children undergoing acute DENV infection (n=97) **(B)**. The expression of IFN-related genes FOXO3 and IRF7 was quantified by RT-qPCR and reported as 2^-ΔCt^ values in PBMCs isolated from healthy donors (n=41) and hospitalized children undergoing acute DENV infection (n=97) **(C)**. Plasma concentrations of IFNα and IFNβ were determined using single molecule assay (Simoa) digital ELISA in healthy donors (n=43) and DENV patients (n=95) **(D)**. Association of plasma concentrations of IFNα and IFNβ with the expression of IFNα and IFNβ genes in DENV patients (n=87) as determined by Spearman’s correlation. All p-values were calculated using Mann-Whitney U test (*p< 0.05; ***p<0.001; ****p<0.0001).

**Figure 2:**
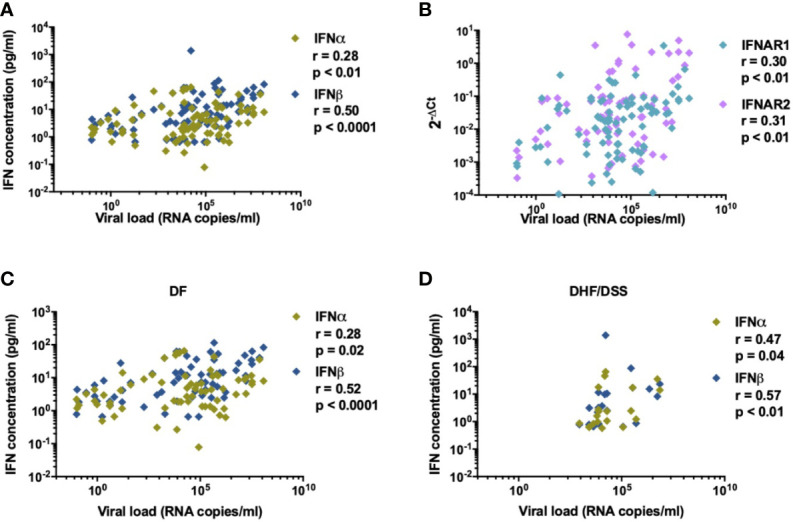
IFN-I is correlated to DENV viral load. Association of **(A)** plasma concentrations of IFNα and IFNβ (n=87) and **(B)** relative expression of IFN-related genes for IFNAR1 and IFNAR2 (n=84) with viral load in plasma from DENV patients was determined using Spearman’s correlation **(C, D)**. Correlation of DENV viral load (RNA copies/ml) with plasma concentrations of IFNα in patients with DF (n=76) and DHF/DSS (n=22).

It remains controversial as to whether IFN-I responses are protective or contribute to immunopathogenesis during DENV infection (39). Detection of IFN-I responses are challenging since in healthy donors and during viral infections only trace amounts of IFN-I mRNA are present in PBMC and determination of protein concentrations in plasma by conventional methods has proven to be unreliable (40–44). We performed gene expression analysis of a set of IFN-I related genes [IFNα, IFNβ, IFN-I receptor subunits (IFNAR1 and IFNAR2), IRF7 and FOXO3] in PBMCs isolated from DENV-infected patients with variable disease severity and healthy donors. Increased transcription of IFNAR2 and IRF7 mRNA were observed in DENV positive patients (IFNAR2: p=0.0006; IRF7: p <0.0001, **Figures 1A, B**) compared to age and gender matched healthy donors. In parallel, we analyzed protein concentrations of IFNα and IFNβ in plasma from by ultra-sensitive single-molecule array (Simoa) digital ELISA technology in the same cohort (35). Here, we observed enhanced protein concentrations of IFNα and IFNβ in acute DENV-infected patients versus healthy donors (IFNα: p=<0.0001 and IFNβ: p=0.0138) (**Figure 1C**). Whereas IFNβ protein concentrations were correlated with IFN*β* mRNA (r=0.35; p<0.01), no such correlation was observed for IFNα (r=0.10; p=0.42) during acute DENV infection (**Figure 1D**). DENV viral load positively correlated with levels of plasma IFNα/β (r=0.28; p<0.01 and r=0.50; p<0.0001) (**Figure 2A**), IFNAR1-mRNA (r=0.30; p<0.01), and IFNAR2-mRNA (r=0.31; p<0.01) (**Figure 2B**). Furthermore, when patients were stratified based on disease severity, DENV viral load correlated positively with plasma IFNα/β in patients with DF (r=0.28; p=0.02 and r=0.52; p<0.0001) and DHF/DSS (r=0.47; p=0.04 and r=0.57; p<0.01) (**Figures 2C, D**). Evaluating the different infecting serotypes, we observed that the IFNα and IFNβ plasma concentrations are similar across all DENV serotypes included in the cohort (DENV-1, DENV-2, DENV-4) (**Supplementary Figure 2**). Taken together, these data show that an elevated viral load is correlated to an increase in IFNα/β protein synthesis and downstream induction of expression of IFN-I related genes (IFNAR2, IRF7) during acute DENV infection.

### Frequencies of pDC Correlate With IFNα Concentration During Acute Dengue Infection

**Figure 3:**
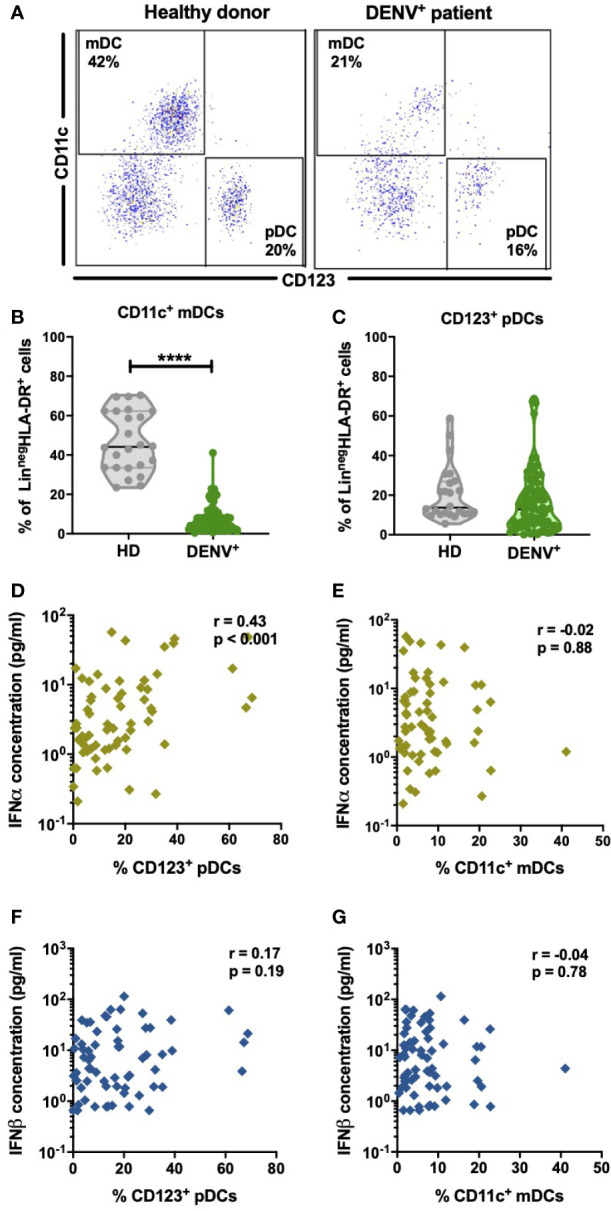
Distribution of dendritic cell subsets in DENV patients and effect on IFN-I **(A)**. Representative dot plot of PBMCs isolated from one healthy child and a hospitalized child undergoing acute DENV infection and stained for dendritic cell subsets based on expression of CD11c and CD123 **(B,C)** Comparison of frequencies of CD123^+^ pDCs and CD11c^+^ mDCs in healthy donors (n=24) and hospitalized children undergoing acute DENV infection (n=77). Data are reported as percentage of Lineage^neg^HLA-DR^+^ cells **(D–G)**. Association of plasma concentrations of IFNα and IFNβ with the frequencies of CD11c^+^ mDCs and CD123^+^ pDCs in DENV patients (n=90) was determined by Spearman’s correlation. All p-values were calculated using Mann-Whitney U test (****p < 0.0001).

pDC and virus-infected cells are the major sources of IFN-I. DENV has tropism for dendritic cells of the myeloid lineage in blood and therefore will contribute to the IFN-I response (25, 45–47). Hence, we aimed to evaluate if changes in circulating frequencies of mDCs and pDCs could account for the changes in IFN-I observed during acute DENV-infection. The gating strategy used for the identification of dendritic cell subsets is outlined in **Supplementary Figure 3**. A representative dot plot showing the gating strategy of CD11c^+^ mDCs and CD123^+^ pDCs in healthy donors and DENV patients is shown in **Figure 3A**. The percentages of CD11c^+^ mDCs were found to be significantly decreased in dengue patients compared to healthy controls (median: 5.4% versus 44.1%; p<0.0001) (**Figure 3B**) whereas no difference was observed in the relative proportions of pDCs between dengue patients and healthy donors (**Figure 3C**). There was no correlation between the percentages of CD11c^+^ mDCs or CD123^+^ pDCs respectively with viral load in all patients or patients stratified for disease severity (**Supplementary Figure 4**). However, of major interest, increased percentages of circulating CD123^+^ pDC positively correlated to increased IFNα plasma protein concentrations in DENV-infected patients (r=0.43; p<0.001) (**Figure 3D**) whereas CD11c^+^ mDC frequencies were not correlated with IFNα plasma concentrations (**Figure 3E**). No correlation was observed between CD11c^+^ mDC or CD123^+^ pDC frequencies and IFNβ (**Figures 3F, G**).

### Influence of Dengue Immune Status on the IFN-I Response

**Figure 4:**
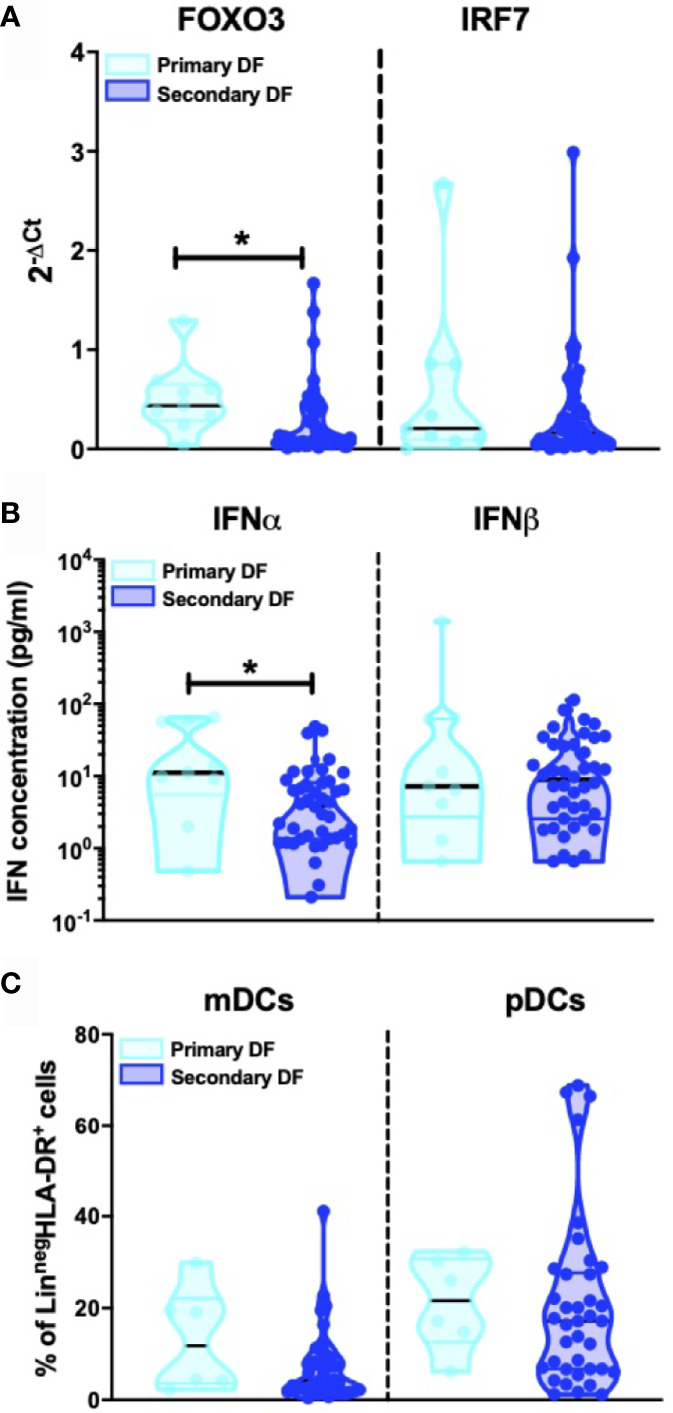
Influence of patient DENV infection history on IFN-I production **(A)**. Expression of IFN related gene FOXO3 and IRF7 was quantified by RT-PCR in PBMCs from DENV patients and patients were classified based on primary (n=9) and secondary DENV infection (n=49) relative to β-glucuronidase (housekeeping gene) **(B)**. Plasma concentrations of IFNα and IFNβ were quantified by Simoa digital ELISA in DENV patients with primary (n=9) and secondary infection (n=42) **(C)**. PBMCs were isolated from hospitalized children undergoing acute primary (n=6) and secondary DENV infection (n=38) and stained for dendritic cell subsets based on expression of CD11c and CD123. All p-values were calculated using Mann-Whitney U test (*p < 0.05).

Heterologous secondary infection increases the risk of a more severe outcome after DENV infection, which may result in DHF or DSS (5, 6, 48). The exact mechanism remains unknown, but an unbalanced and excessive immune response to the infecting serotype seems to contribute to disease severity (49). Hence, we compared levels of IFN-I related genes, IFNα/β protein concentrations and DC subsets between primary infected DF patients (n=9) and secondary infected DF patients (n=49). Here, a decrease in FOXO3-mRNA levels in patients with secondary infection compared to primary infection was observed (p=0.015) whereas levels of other IFN-I related genes were similar in primary infected DF patients compared to secondary infected DF patients (**Figure 4A** and data for IFN-α2, IFN-β, IFNAR1, and IFNAR2 not shown). Individuals undergoing a secondary DF infection showed decreased IFNα protein concentrations compared to primary infected patients (p=0.016) (**Figure 4B**). As shown in **Figure 4C**, the percentages of circulating CD11c^+^ mDCs and CD123^+^ pDC were not different between primary and secondary DF. Taken together, these data show that the type I IFN response is modulated by the infection history of the patients.

### Decreased Expression of IFN-I During the Acute Phase of Dengue Infection Is Associated With Severe Dengue Disease

**Figure 5:**
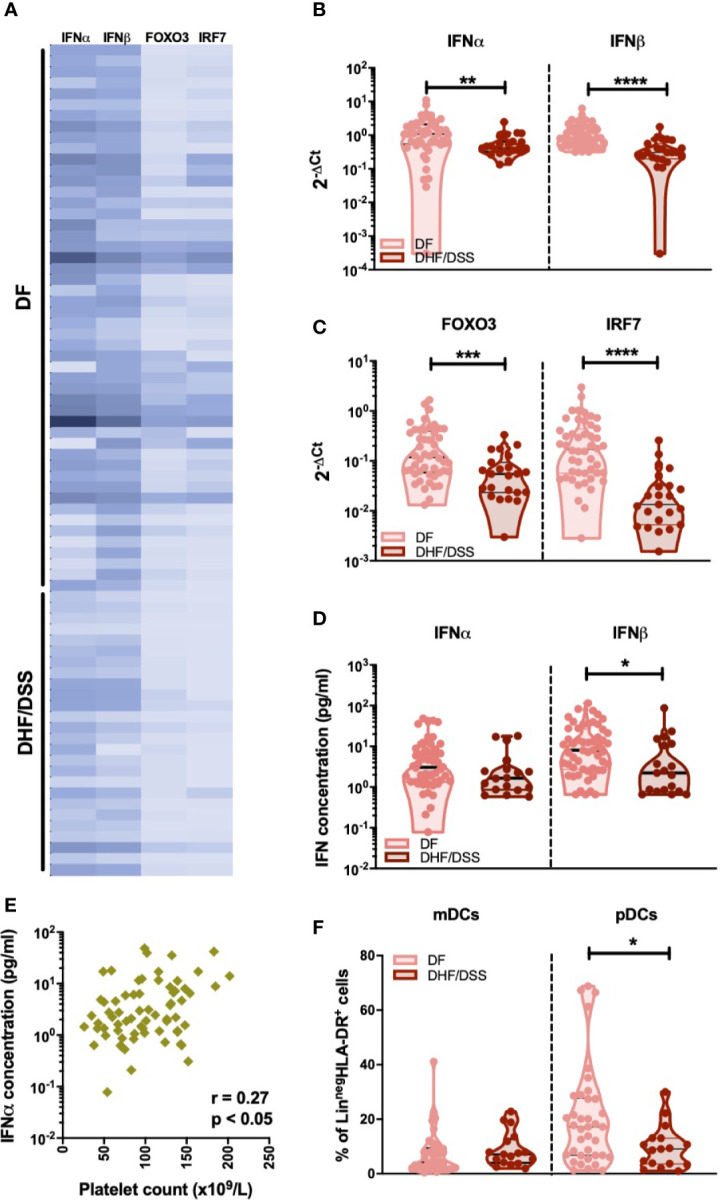
Association of IFNα/β production with infection severity in DENV patients **(A)**. Expression of IFNα/β and IFN related genes FOXO3 and IRF7 in children undergoing acute secondary DENV infection with DF (n=50) and DHF/DSS (n=26) is represented with a heat map **(B)**. Expression of IFNα and IFNβ in patients with secondary infection and classified as DF (n=48) and DHF/DSS (n=23) **(C)** Expression of IFN related genes FOXO3 and IRF7 in patients with secondary infection and classified as DF (n=48) and DHF/DSS (n=22) **(D)** Plasma concentrations of IFNα and IFNβ were quantified by Simoa digital ELISA in DENV patients undergoing secondary infection with DF (n=54) and DHF/DSS (n=19) **(E)** Association of plasma concentrations of IFNα in DENV patients (n=68) with the respective platelet counts (x10^9^/L) **(F)**. Comparison of frequencies of CD11c^+^ mDCs and CD123^+^ pDCs in DENV patients with secondary infection and classified as DF (n=38) and DHF/DSS (n=17). Data is reported as percentage of Lineage^neg^HLA-DR^+^ cells. All p-values were calculated using Mann-Whitney U test (*p < 0.05; **p < 0.01, ***p < 0.001; ****p < 0.0001).

Few and conflicting data are available on the IFN-I response in severe dengue cases (15–19). Thus, we compared mRNA levels of IFN-I genes in DENV infected children who developed classical DF (n=50) with patients classified as DHF or DSS (n=26). As we observed minor differences in IFN-I responses in primary versus secondary infected DF patients, we only included secondary infected patients for this analysis. Reduced expression of IFNα (p<0.001), IFNβ (p<0.0001), IRF-7 (p<0.0001), and FOXO3 (p<0.001) transcripts were observed in patients with severe dengue (DHF/DSS patients) than patients with non-severe manifestations (DF patients) (**Figures 5A–C**). In accordance to the gene expression data, plasma protein concentrations of IFNα (p=0.09) and IFNβ (p=0.013) were lower in DSS/DHF patients compared to DF patients (**Figure 5D**). A decrease in platelet counts is one of the hallmarks of severe DENV infection and is used in the WHO classification criteria (30). In accordance with the observation of a decreased IFN-I response in severe disease, we observed that lower platelet counts are correlated to lower IFNα protein plasma concentrations in DENV-infected patients (p<0.05, r = 0.27, **Figure 5E**). As frequencies of circulating pDCs are associated to IFNα protein concentration during DENV infection (**Figure 3D**), we examined the influence of dengue disease severity in patients with secondary infection on the distribution of dendritic cell subsets. A significant decrease in the relative proportions of pDCs (median: 17.2% vs. 9.1%; p<0.05), but not mDCs (**Figure 5F**) was observed in secondary DHF/DSS compared to secondary DF cases. Hence, decreased pDC frequencies and an associated decreased IFN-I response are observed in patients developing severe disease during secondary DENV infection, indicating that an early IFN-I response mediated by pDCs is beneficial for clinical outcome after DENV infection.

### IFN-I Response Correlates With Anti-DENV Antibody Titers During Secondary Acute Dengue Infection

**Figure 6:**
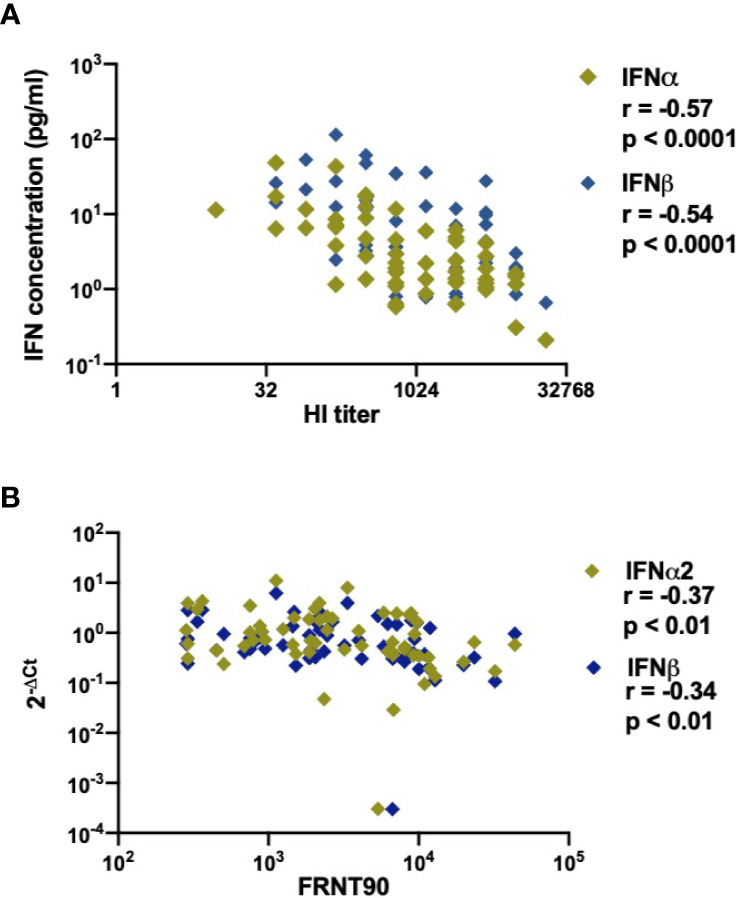
Association of DENV neutralizing antibody titers (FRNT90) in patients with expression and production of IFNα/β plasma concentrations and gene expression **(A)**. Correlation of plasma concentrations of IFNα & IFNβ in DENV patients (n=58) with respective hemagglutination inhibition assay (HI) titers measured at discharge from hospital **(B)**. Correlation of expression of IFNα/β genes in DENV patients (n=59) with respective FRNT90 titers measured at discharge from hospital.

As IFN-I play an important role in the induction of the humoral immune response during infection or vaccination (50, 51), we sought to determine the relationship between type I IFN and development of anti-DENV antibodies in infected patients. As previous infection history has a major impact on the developing antibody titers during the acute phase of DENV infection (52), we included only secondary infected patients for this analysis. Functional neutralizing antibodies were measured by the focis reduction neutralization test (FRNT90) (34). Even though their presence does not always correlate with protection from severe disease (53), the assay remains the gold standard for measuring humoral protection during DENV infection. Antibody titers were measured at discharge in order to reach sufficient titers for detection. Of interest, lower concentrations of IFNα and IFNβ proteins correlated with higher total anti-dengue antibodies measured at hospital discharge by hemagglutinin inhibition (HI) assay (p<0.0001, r=-0.57 and p<0.0001, r =-0.54) (**Figure 6A**). In parallel, lower IFNα (r=-0.37; p<0.01), IFNβ (r =-0.34; p <0.01), IFNAR1 (r=-0.27; p<0.05), and IRF7 (r=-0.28; p<0.05) transcripts measured at hospital admittance correlated with higher FRNT90 titers at hospital discharge (**Figure 6B**, **Supplementary Figures 5A, B**). Of note, these correlations were independent of the amount of days between hospital admittance and discharge (**Supplementary Figure 6**). As type I IFN correlates with viral load (**Figure 2**) and is dependent on disease severity (**Figure 5**), we also analyzed the correlation of neutralizing antibodies to viral load. Here, neutralizing antibody titers did not correlate to viral load (**Supplementary Figure 7**) in DF or DHF/DSS patients. Taken together, we observed strong negative correlations between IFN-I response measured at hospital admittance and the development of anti-DENV antibody titers in our cohort of acute DENV-infected Cambodian children.

## Discussion

Here, we present a thorough investigation of the IFN-I response during the acute phase of DENV infection in a cohort of 115 DENV-infected patients. All patients were included at early onset of symptoms (< 96 h of fever), were well characterized virologically and classified for dengue disease severity according to WHO 1997 criteria at discharge. This allows us to stratify for immune status and disease severity, limiting the influence of confounding factors in our analysis. Our study demonstrates a protective function of an early and robust IFN-I response, as measured both by gene expression and plasma protein concentrations, from the development of DHF/DSS after DENV infection. Lower platelet counts, indicative of more severe disease, were associated with lower IFNα plasma concentrations.

Previous studies analyzing circulating protein concentrations of IFNα/β during DENV infection are conflicting, partly due to the low sensitivity of the assays used (15–19, 54, 55). By using a Single-molecule array (Simoa) digital ELISA, we were able to analyze the amount if IFN-Is quantitatively in plasma samples and to detect femtomolar concentrations especially of IFNα. In addition, most gene expression data for IFN-I related genes are derived from microarray or next-generation sequencing experiments which are less specific and sensitive than quantitative PCR using a primer/probe combination (56, 57).

IRF7 is a main transcriptional factor downstream of RLRs/TLRs signaling, and promotes IFN-I response induction during DENV infection (9–11). Here, we observed a robust increase in IRF7 in DENV-infected patients compared to healthy donors, and a lower expression in severe DHF/DSS patients compared to mild DF patients. FOXO3 on the other hand antagonizes signaling intermediates downstream of TLR4, NF‐κB and IRFs, resulting in inhibition of IFNβ expression (58). We detected a lower expression of FOXO3 in secondary DENV-infected individuals compared to primary infected individuals and in severe DHF/DSS patients compared to mild DF patients. One explanation could be that FOXO3 transcription might be decreased to avoid an excessive down modulation of IFN responses in a negative feedback loop (58).

We also investigated the expression of the IFNα/β receptors, IFNAR1, and IFNAR2 during DENV infection. We observed an increased expression of IFNAR2 transcript in DENV-infected individuals, which correlated with viral loads. Soluble IFNAR2 detected in serum, saliva, urine, and the peritoneal fluid of both humans and mice (59, 60), can have a carrier function for IFN-I increasing their stability and enhancing their biological activity (61). In addition, IFNAR2 can also repress transcription of the IFN-stimulated response elements reporter, thus modulating gene transcription of all IFN-activated cellular genes (62). IFNAR1 has been reported as a short‐lived protein in different cell types (63, 64) and its expression is lower than that of IFNAR2 and is correlated to desensitization of IFNα in mature DCs (65).

Notably, plasma IFNα and IFNβ concentrations were also elevated in DENV patients and were associated with higher viral load. Detected IFNβ protein concentrations were moderately correlated to IFNβ transcripts, however, no such correlation was observed for IFNα. The synthesis of IFNα/β is regulated at the posttranscriptional level (66) and the mRNA transcripts are highly unstable. It has been shown that the rapid decay of IFNα and IFNβ transcripts correlated with a large number of the AU-rich element (ARE)-containing 3′ untranslated regions (3′-UTRs), which are known to destabilize mRNA (67). This can explain the differences observed between the determination of mRNA levels in PBMCs and protein concentrations in the plasma (68).

We observed a lower IFNα protein concentration in secondary infection compared to primary infection, in accordance with previous published data (16, 18, 19). However, IFNβ protein concentration was similar irrespective of infection history. IFNα binds IFNAR1 with a lower affinity as compared to IFNβ (69) and this could explain why sustained signaling appears possible by IFNβ but not IFNα2 (70). The IFNAR1–IFNβ complex has been also shown to transduce signals that modulated expression of a distinct set of genes independently of Jak-STAT pathways (71). Of note IFNα has been reported to be involved in the control of LCMV spread, as only blockade of IFNα, but not IFNβ affects early viral dissemination (72).

Type I IFN plasma concentrations and IFNAR1/2 transcripts are positively correlated to viral load, which is in line with previous studies in DENV and chronic HIV and emphasize the role of type I IFN in the control of viral load during infection (15, 19, 73, 74).

A limited number of studies have described the frequencies of circulating mDCs and pDCs in dengue-infected individuals (27, 28). The decrease in peripheral mDCs in DENV-infected patients and reduction in pDCs in severe dengue patients could be explained by several non-exclusive mechanisms: a direct viral cytopathic effect on DCs, migration of DCs to secondary lymphoid organs or virus/cytokine-driven dysfunction of bone marrow resulting in a lower production of DCs (28). In general, pDCs and virus-infected mDCs can be major sources of IFN-I. It is known that pDCs are stimulated upon physical contact with DENV infected cells, during which viral RNA is sensed by pDCs, leading to antiviral response activation through TLR7 (22, 75). Our data shows an association between IFNα plasma concentration and the percentage of circulating pDCs, indicating that these, and not mDC might account for the type I IFN production in the acute phase of DENV infection. In DHF/DSS patients, frequencies of circulating pDCs, not mDCs and type I IFN responses are decreased compared to DF patients. Therefore, induction of a robust IFN-I response, possibly by pDCs, rather than by virus-infected mDCs, seems to protect the host from the development of severe disease. In accordance, we also demonstrated that increased IFN-I expression was detected in patients with a milder disease phenotype after dengue infection. Furthermore, we observed a correlation between IFNα protein concentration and platelet counts.

It is known that during acute viral infections, IFN-Is exert their activity directly on B cells or by stimulating B cell responses through DCs or CD4^+^ T cells resulting in B cell activation, production of neutralizing antibodies and isotype class switching (48, 49, 76–78). In agreement, a correlation between type I IFN responses and anti-DENV antibody titers was observed. However, the precise mechanism behind this observation remains to be investigated further.

Overall, our study indicates that IFN-I produced in the acute phase of infection is associated with less severe outcome of dengue disease. Novel detection methods for IFN-I might help in the stratification of patients at hospital admittance.

## Data Availability Statement

The original contributions presented in the study are included in the article/**Supplementary Materials**; further inquiries can be directed to the corresponding author/s.

## Ethics Statement

The studies involving human participants were reviewed and approved by the National Ethics Committee for Health Research, Cambodia. Written informed consent to participate in this study was provided by the participants’ legal guardian/next of kin.

## Author Contributions

TC and CS conceived the project, designed the study, selected the samples, analyzed and interpreted the data, and wrote the manuscript. VU conducted the experiments, performed the data analysis, and prepared the manuscript. SLy, DL, and SH coordinated to the recruitment of the dengue patients and healthy controls. FF, NS, VB, AV, SLa, HA, and DD conducted the experiments and performed the data analysis. VD, PD, and TC classified the dengue patients. NS, HA, VD, PD, and DD revised the manuscript. All authors contributed to the article and approved the submitted version.

## Funding

This study was supported by a grant from Institut Pasteur (ACIP n°221 -2019; DeSiReS: Dengue Severe Immune Response Studies). TC was funded by the Institute Pasteur International Network and is an HHMI-Wellcome Trust International Research Scholar (208710/Z/17/Z). VU was funded by the Institute Pasteur International Network Calmette and Yersin Ph.D. scholarship. DD acknowledges support from the Agence Nationale de la Recherche (ANR grant number CE17001002).

## Conflict of Interest

The authors declare that the research was conducted in the absence of any commercial or financial relationships that could be construed as a potential conflict of interest.

## Supplementary Material

Supplementary Figure 1 Patient characterization **(A)** Schematic representation of the work-flow in PBMCs and plasma samples from healthy donors and DENV patients. PBMCs were isolated by Ficoll density gradient centrifugation and divided for **i)** phenotyping of dendritic cell subsets by flow cytometry and **ii)** RT-qPCR for determining expression of IFNα/β and IFN-I related genes. **iii)** IFNα/β concentrations were determined in plasma samples from healthy donors and DENV patients using ultrasensitive digital ELISA (**B**). Comparison of DENV viral load (RNA copies/ml) between patients with DF (n=80) and DHF/DSS (n=26).

Supplementary Figure 2 Influence of infecting DENV serotype on IFN-I production. Comparison of plasma concentrations of IFNα and IFNβ in patients infected with DENV-1 (n=30), DENV-2 (n=53), and DENV-4 (n=5). All groups were compared using Kruskal-Wallis test.

Supplementary Figure 3 Representative gating strategy to define dendritic cell subsets. PBMCs were stained for dendritic cell markers and gated on both lymphocytes and monocytes followed by doublet discrimination. Lineage^-^HLA-DR^+^ were selected **(A)** and gated as CD11c^+^ myeloid dendritic cells **(B1)** and CD123^+^ plasmacytoid dendritic cells **(B2)**.

Supplementary Figure 4 Association of frequencies of dendritic cells with viral load. Correlation of DENV viral load (RNA copies/ml) with frequencies of CD11c^+^ mDCs and CD123^+^ pDCs in patients with DF and DHF/DSS.

Supplementary Figure 5 Association of neutralizing antibody titers (FRNT90) in DENV patients with expression of IFN-I related genes (**A**). Correlation of expression of IFN receptors IFNAR1 and IFNAR2 in DENV patients (n=59) measured at hospital admittance with respective FRNT90 titers at hospital discharge (**B**). Correlation of expression of IFN related genes FOXO3 and IRF7 measured at hospital admittance in DENV patients (n=56) with respective FRNT90 titers measured at hospital discharge.

Supplementary Figure 6 Association of antibody titers with IFN-I production in dengue patients based on duration of hospitalization. Correlation of plasma concentrations of IFNα and IFNβ obtained at hospital admission with patient HI titers measured at discharge, stratified based on the length of hospital stay {difference between day of admission and day of discharge from hospital [2 days (n=23), 3 days (n=19), 4 days (n=10), 5 days (n=5)]}.

Supplementary Figure 7 Association of neutralizing antibody titers with viral load. Correlation of DENV viral load (RNA copies/ml) with respective FRNT90 titers at hospital discharge in dengue patients with DF (n=39) and DHF/DSS (n=16).
