# Supplementary figures and images for: Decreased Type I Interferon Production by Plasmacytoid Dendritic Cells Contributes to Severe Dengue
Source: Front Immunol. 2020 Dec 17;11:605087. doi: 10.3389/fimmu.2020.605087 (PMC7773824; doi:10.3389/fimmu.2020.605087)

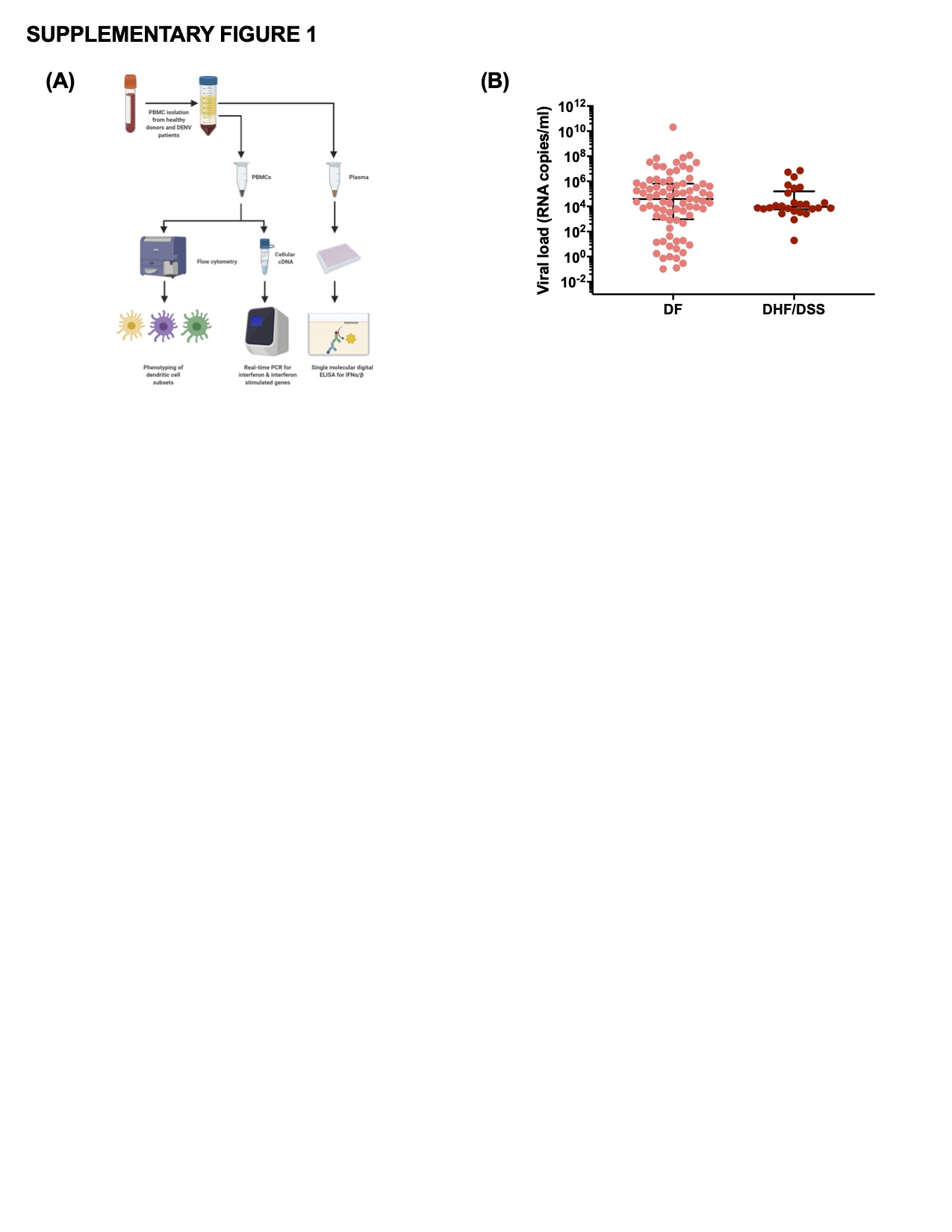

Supplement: Supplementary Figure 1 — Patient characterization (A) Schematic representation of the work-flow in PBMCs and plasma samples from healthy donors and DENV patients. PBMCs were isolated by Ficoll density gradient centrifugation and divided for i) phenotyping of dendritic cell subsets by flow cytometry and ii) RT-qPCR for determining expression of IFNα/β and IFN-I related genes. iii) IFNα/β concentrations were determined in plasma samples from healthy donors and DENV patients using ultrasensitive digital ELISA (B). Comparison of DENV viral load (RNA copies/ml) between patients with DF (n=80) and DHF/DSS (n=26). [file Image_1.jpeg]

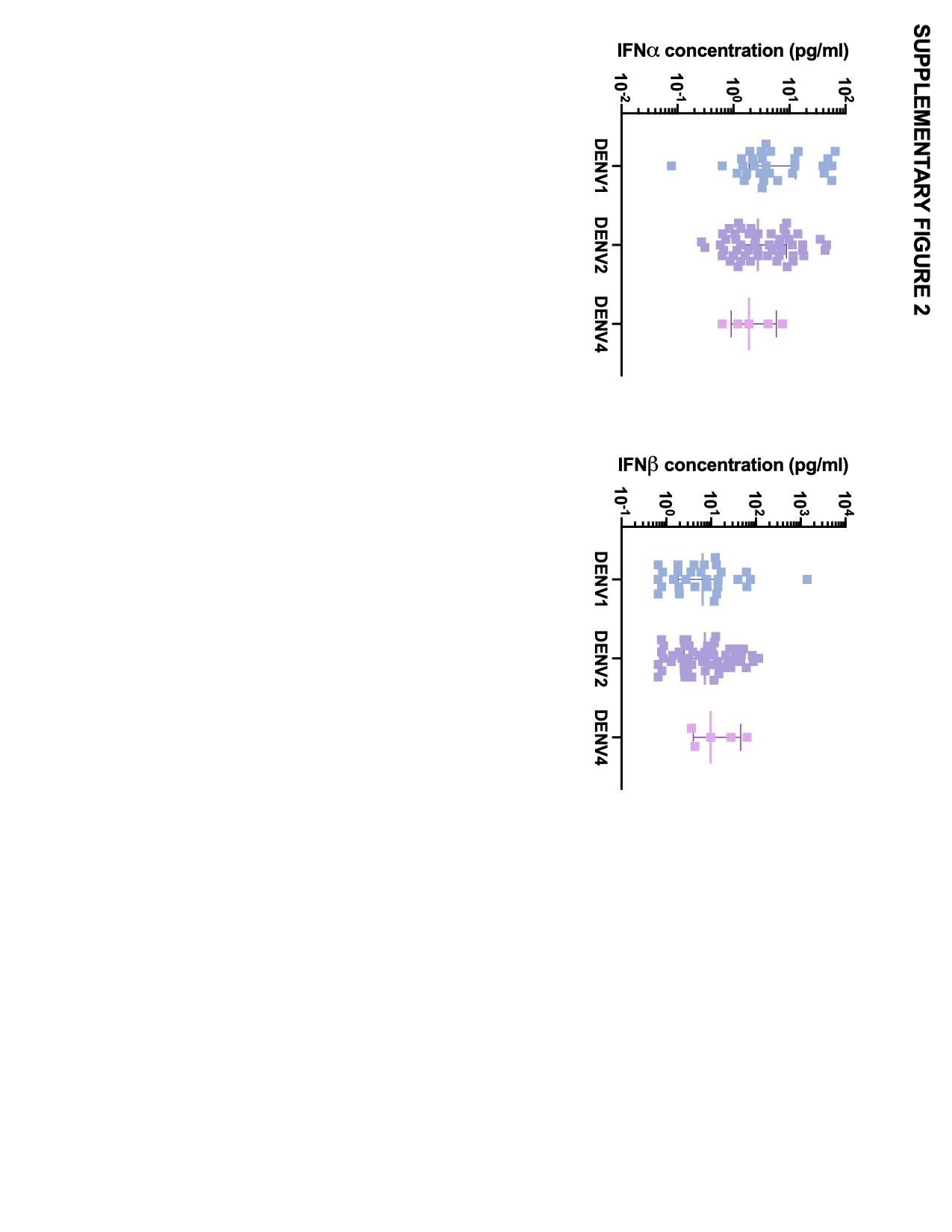

Supplement: Supplementary Figure 2 — Influence of infecting DENV serotype on IFN-I production. Comparison of plasma concentrations of IFNα and IFNβ in patients infected with DENV-1 (n=30), DENV-2 (n=53), and DENV-4 (n=5). All groups were compared using Kruskal-Wallis test. [file Image_2.jpeg]

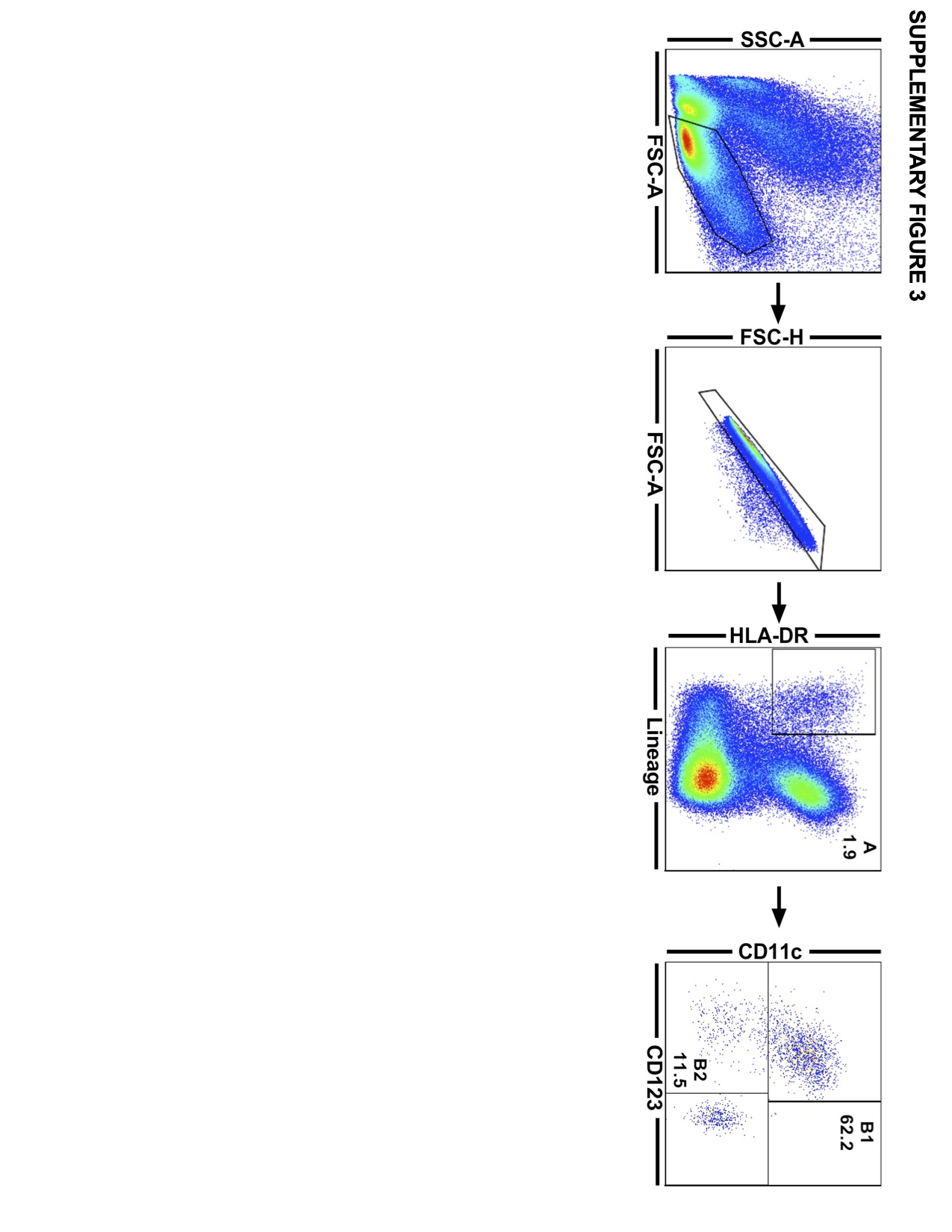

Supplement: Supplementary Figure 3 — Representative gating strategy to define dendritic cell subsets. PBMCs were stained for dendritic cell markers and gated on both lymphocytes and monocytes followed by doublet discrimination. Lineage-HLA-DR+ were selected (A) and gated as CD11c+ myeloid dendritic cells (B1) and CD123+ plasmacytoid dendritic cells (B2). [file Image_3.jpeg]

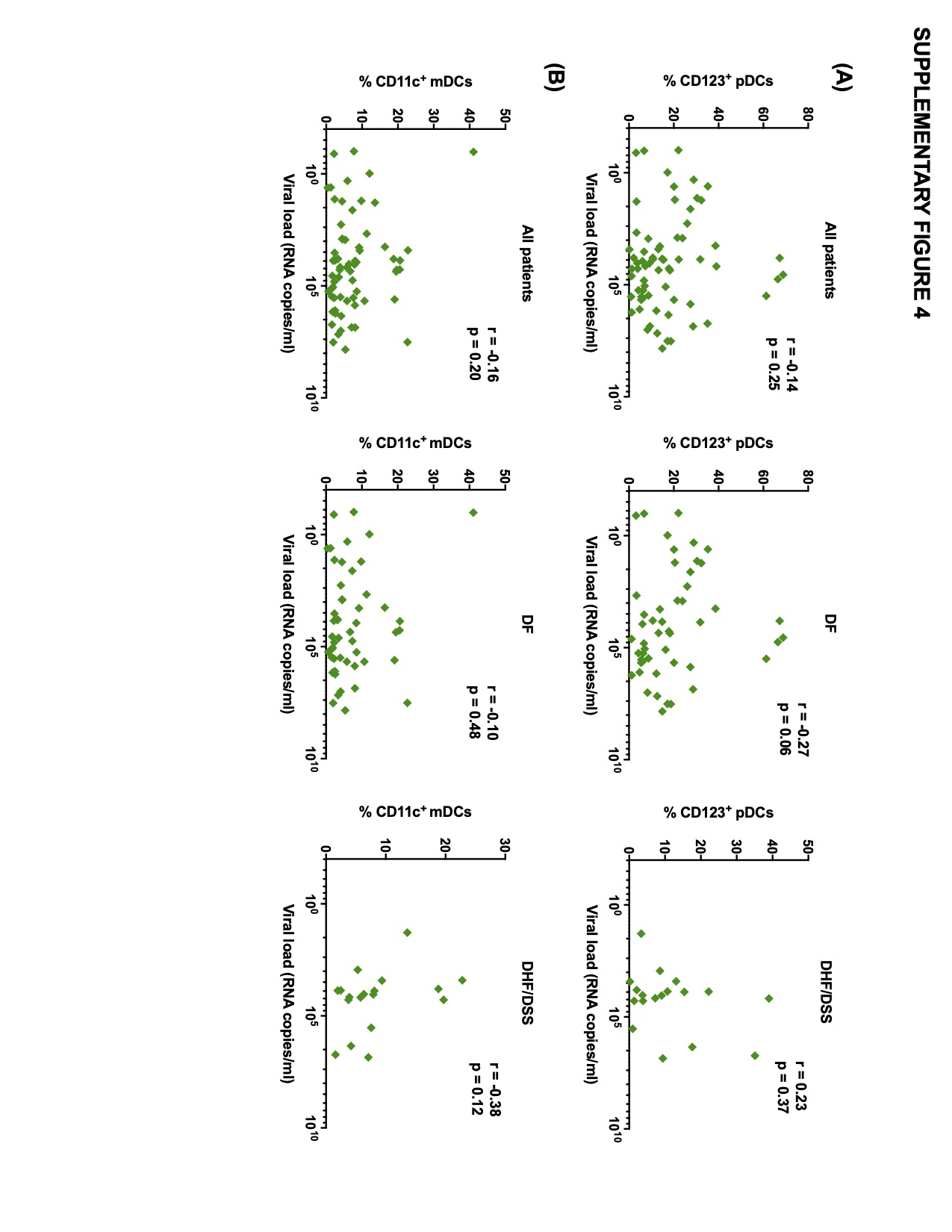

Supplement: Supplementary Figure 4 — Association of frequencies of dendritic cells with viral load. Correlation of DENV viral load (RNA copies/ml) with frequencies of CD11c+ mDCs and CD123+ pDCs in patients with DF and DHF/DSS. [file Image_4.jpeg]

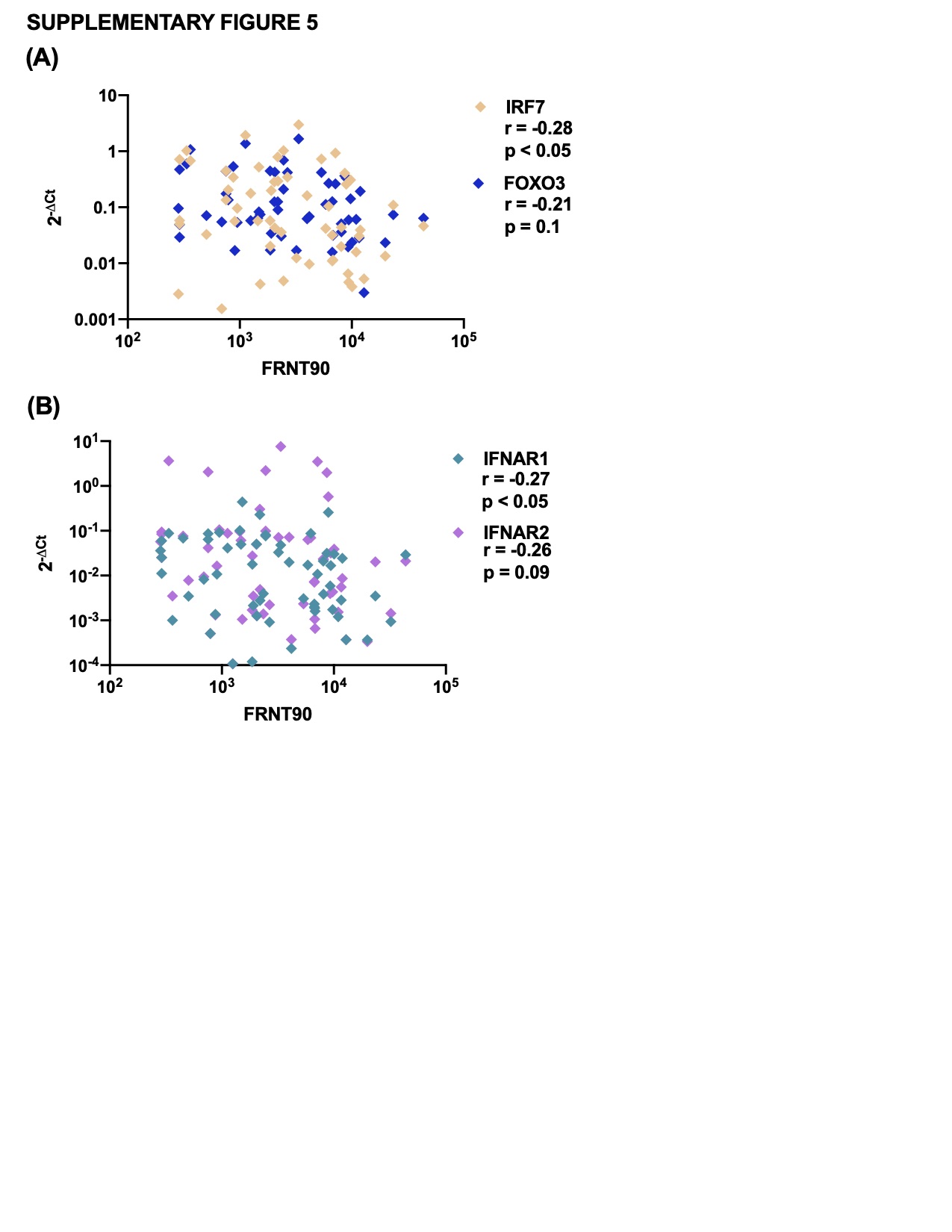

Supplement: Supplementary Figure 5 — Association of neutralizing antibody titers (FRNT90) in DENV patients with expression of IFN-I related genes (A). Correlation of expression of IFN receptors IFNAR1 and IFNAR2 in DENV patients (n=59) measured at hospital admittance with respective FRNT90 titers at hospital discharge (B). Correlation of expression of IFN related genes FOXO3 and IRF7 measured at hospital admittance in DENV patients (n=56) with respective FRNT90 titers measured at hospital discharge. [file Image_5.jpeg]

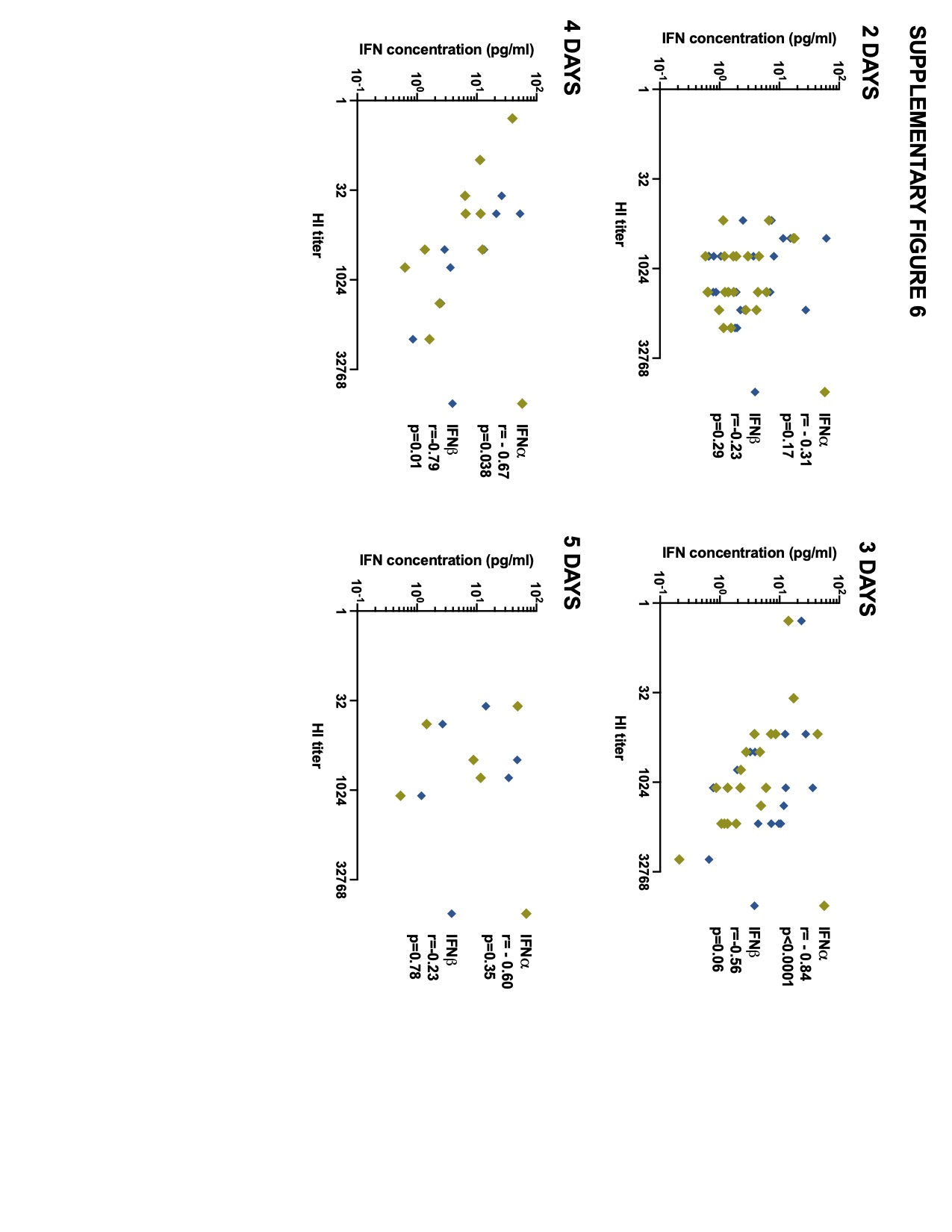

Supplement: Supplementary Figure 6 — Association of antibody titers with IFN-I production in dengue patients based on duration of hospitalization. Correlation of plasma concentrations of IFNα and IFNβ obtained at hospital admission with patient HI titers measured at discharge, stratified based on the length of hospital stay {difference between day of admission and day of discharge from hospital [2 days (n=23), 3 days (n=19), 4 days (n=10), 5 days (n=5)]}. [file Image_6.jpeg]

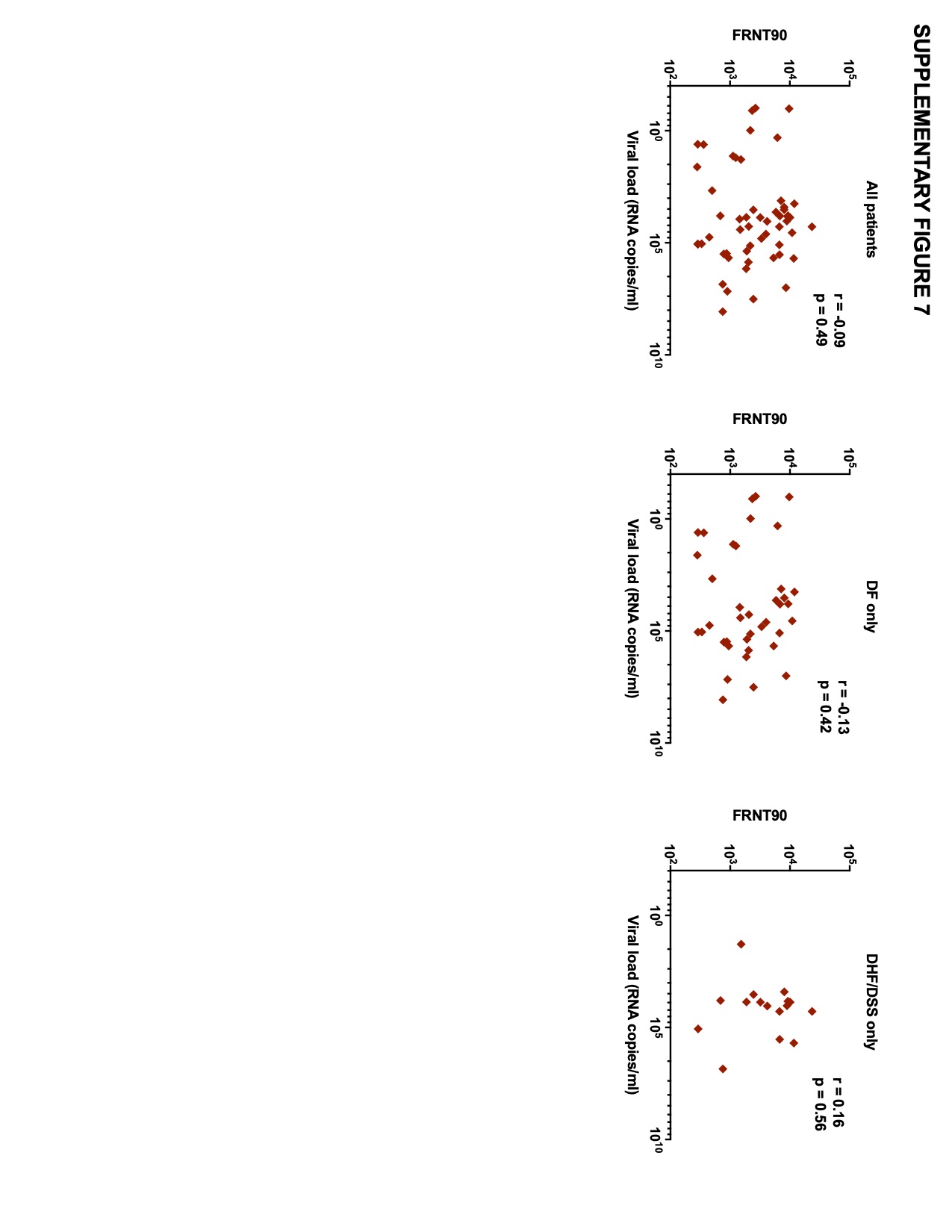

Supplement: Supplementary Figure 7 — Association of neutralizing antibody titers with viral load. Correlation of DENV viral load (RNA copies/ml) with respective FRNT90 titers at hospital discharge in dengue patients with DF (n=39) and DHF/DSS (n=16). [file Image_7.jpeg]
